# Risk stratification for advanced colorectal neoplasia based on the findings of the index and first surveillance colonoscopies

**DOI:** 10.1371/journal.pone.0245211

**Published:** 2021-01-22

**Authors:** Munenori Honda, Hideaki Naoe, Ryosuke Gushima, Hideaki Miyamoto, Masakuni Tateyama, Kouichi Sakurai, Yasushi Oda, Yoshitaka Murakami, Yasuhito Tanaka

**Affiliations:** 1 Department of Gastroenterology and Hepatology, Kumamoto University Hospital, Kumamoto, Japan; 2 Hattori Clinic, Kumamoto, Japan; 3 Oda GI Endoscopy and Gastroenterology Clinic, Kumamoto, Japan; 4 Department of Medical Statistics, Toho University, Tokyo, Japan; University Medical Center of Princeton at Plainsboro, UNITED STATES

## Abstract

Risk stratification by index colonoscopy is well established for first surveillance endoscopy, but whether the previous two colonoscopies affect the subsequent advanced neoplasias has not been established. Therefore, the subsequent risk based on the findings of the index and first surveillance colonoscopies were investigated. This retrospective, cohort study was conducted in two clinics and included participants who had undergone two or more colonoscopies after index colonoscopy. High-risk was defined as advanced adenoma (≥ 1 cm, or tubulovillous or villous histology, or high-grade dysplasia). Based on the findings of the index and first surveillance colonoscopies, patients were classified into four categories: category A (both colonoscopy findings were normal), category B (no high-risk findings both times), category C (one time high-risk finding), and category D (high-risk findings both times). The incidence of subsequent advanced neoplasia was examined in each category. A total of 13,426 subjects were included and surveyed during the study periods. The subjects in category D had the highest risk of advanced neoplasia (27.4%, n = 32/117). The subjects in category A had the lowest risk (4.0%, n = 225/5,583). The hazard ratio for advanced neoplasia of category D compared to category A was 9.90 (95% Confidence interval 6.82–14.35, P<0.001). Classification based on the findings of index and first surveillance colonoscopies more effectively stratifies the risk of subsequent advanced neoplasia, resulting in more proper allocation of colonoscopy resources after two consecutive colonoscopies.

## Introduction

Colorectal cancer (CRC) is the third leading cause of morbidity and the fourth leading cause of cancer-related death in the world [1]. Colonoscopy is the main modality for diagnosing CRC. This procedure also has the potential to prevent CRC by removing precancerous lesions, and it is thus an important tool to help improve outcomes. To prevent subsequent cancer after the removal of adenomatous polyps, practice guidelines recommend periodic colonoscopy surveillance, which depends on the number of adenomas and whether they are advanced based on size or histology by index colonoscopy. Moreover, current surveillance guidelines have been refined based on updated evidence and the consensus of experts [2–5].

The effect of consecutive colonoscopy findings has been also under discussion. A long-term future risk classification based on the results from two consecutive examinations might help establish more effective evaluation subsequent intervals than one based on the most recent examination.

In our large cohort with a total of 13,426 subjects, the aim of our study was to assess the subsequent incidence of advanced neoplasia (AN, including CRC and advanced adenoma) after the first surveillance colonoscopy based on the findings from the index and first surveillance colonoscopies, i.e. two consecutive colonoscopies and to compare with basis of usual index colonoscopy findings only.

## Methods

### Study participants

This retrospective cohort study involved subjects who underwent colonoscopy at two specialty gastroenterology practice clinics in Kumamoto, Japan. More than 30 endoscopists certified by the Japan Gastroenterological Endoscopy Society performed the colonoscopies. The study was approved by the institutional review board (IRB) of Kumamoto University Hospital (Accession No. 1891), and it conformed to the provisions of the Declaration of Helsinki (as revised in Fortaleza, Brazil, October 2013). All data were obtained with anonymized settings. Each patient who had colonoscopy in daily clinical practice provided non-specific informed consent that data is used for medical research with anonymized setting. Then the IRB waived the requirement for specific informed consent in this study.

The index colonoscopy was defined as the first evaluation between January 2005 and December 2014. Subjects who underwent at least three colonoscopies (an index colonoscopy and two surveillance examinations) performed at the clinics were included. Based on the index colonoscopy, patients were excluded for the following reasons: age < 40 years, CRC that necessitated surgery, history of colectomy, inflammatory bowel disease (IBD), familial adenomatous polyposis (FAP), or other polyposis. The data were collected from the electronic records and included polyp findings (pathology results, size and number) and patient demographics from 2005 through 2019. All data were accessed on October 2019.

Bowel preparation used one of two liquid preparations (polyethylene glycol [PEG] or sodium phosphate). The size and diagnosis of the polyps were determined by the performing endoscopist and confirmed by the pathology report. The colonoscopy was repeated if the bowel preparation was poor. An examination with a follow-up interval of < 6 months was summarized as one procedure. Basically, polyps larger than 5 mm were resected using either polypectomy or endoscopic mucosal resection. However, some diminutive polyps (≤5mm) that did not show features with advanced adenomas by using narrow-band imaging, mainly magnification endoscopy were left unresected according to the decision of the endoscopists according to Japanese colorectal polyp management guideline [6, 7]. Therefore, the number of small polyps including diminutive polyps was not taken into consideration when determining the risk in this study.

Based on the findings of the index colonoscopy, the study population was categorized into three groups: (1) normal group; no adenoma; (2) low-risk group; small adenomas less than 10 mm including unresected diminutive adenoma; and (3) high-risk group; advanced adenomas (AAs), which included cases of an adenoma ≥10 mm, adenoma with tubulovillous or villous histology, adenoma with high-grade dysplasia. Furthermore, to identify the best stratification, the patients were classified into nine groups based on the combination of findings of index colonoscopy and first surveillance colonoscopy. Endoscopically resected serrated lesions were classified according to their histological dysplasia only, as normal, low-risk, or high-risk. When more than one polyp was found, the most advanced one (in terms of size or histology) was used for classification.

### Statistical analysis

The primary endpoint in this study was AN incidence at second surveillance or subsequent colonoscopies. To investigate the usefulness of risk assessment based on the index and first surveillance colonoscopy findings, the subjects were classified into nine groups. AN incidence was compared using Kaplan-Meier curves. Risk factors associated with AN were investigated using a Cox regression model, with age and sex included as risk factors. All P values are two-sided, and P values of less than 0.05 were considered significant. All statistical analyses were performed using SPSS software version 25.0.

## Results

### Enrollment of the subjects in this study (Fig 1)

**Fig 1:**
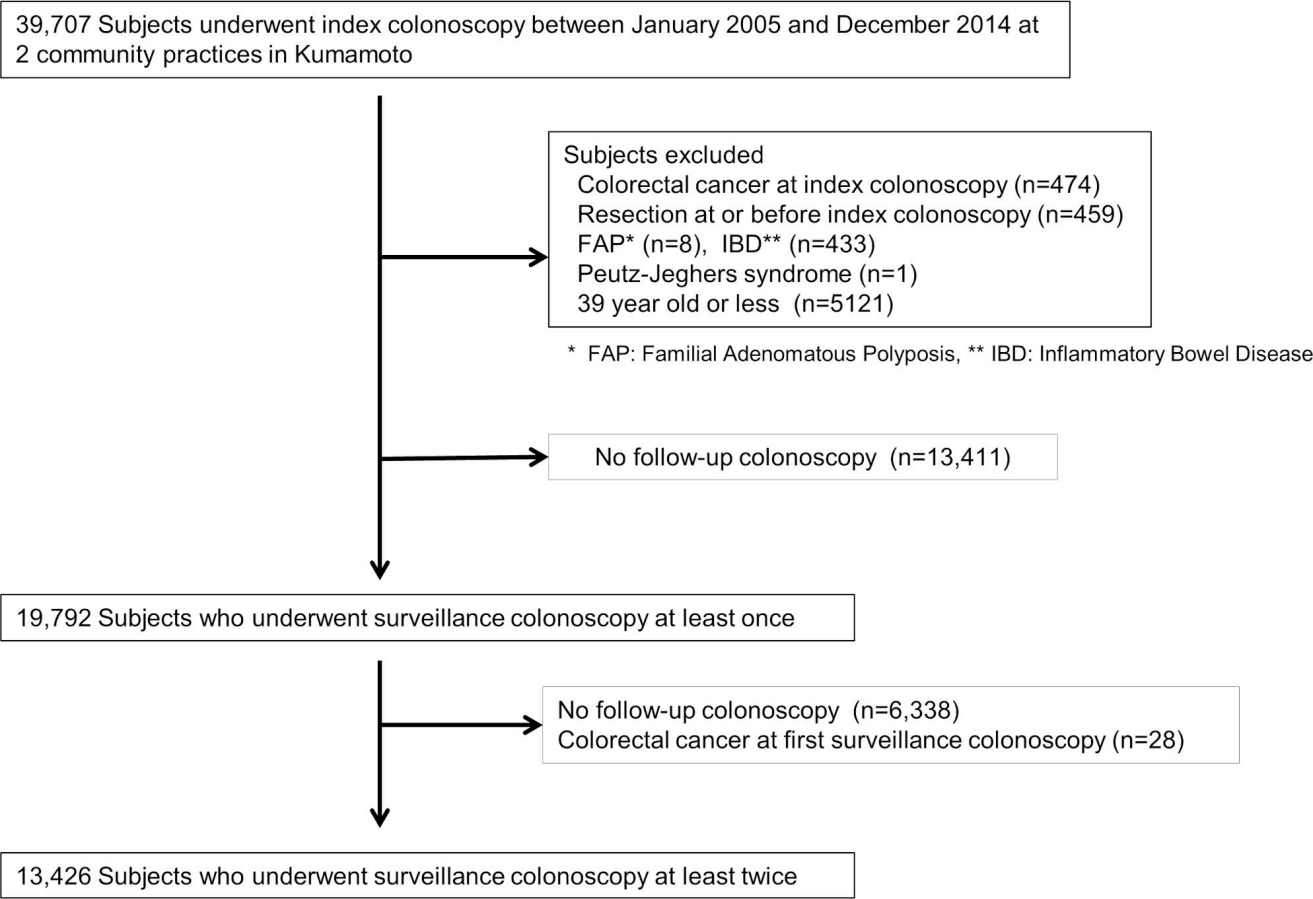
Flowchart of enrollment in this study.

A total of 39,707 participants underwent index colonoscopies at the two clinics. A total of 474 (1.20%) subjects with invasive CRC at index colonoscopy, 459 subjects with a history of colectomy, 433 patients with IBD, 8 patients with FAP, 1 patient with Peutz-Jeghers syndrome and 5,121 subjects who were less than 40 years of age were excluded. Furthermore 13,411 subjects who had no follow up colonoscopy were excluded. Among them, 8,755 subjects (65.3%) had no polyps. Overall, 28 patients among 19,792 subjects (0.14%) were found to have cancer on the first surveillance colonoscopy. Finally, 13,426 subjects who underwent index and at least two surveillance colonoscopies were included in the study.

### Characteristics of the study population and surveillance results based on the index colonoscopy findings

The characteristics of the subjects based on the index colonoscopy findings are shown in Table 1. The mean age at the time of the index colonoscopy was 59.5 ± 9.6 years, and 6,439 (48.0%) were males. The median interval between the index and first surveillance examinations was 1.6 (inter-quartile range (IQR): 1.04–2.76) years, and 10,524 (78.4%) subjects underwent a repeat colonoscopy within 3 years. Participants with higher risk findings at the index colonoscopy tended to have shorter intervals to the first surveillance colonoscopy, 1.81 years at normal group, 1.39 years at low risk, and 1.16 years at high risk, respectively. After the first surveillance examination, 6,660 (49.6%) subjects underwent follow-up more than 5 years later. After first surveillance, the median follow-up period and frequency of colonoscopy were 4.98 (IQR: 2.85–7.94) years and 2 (IQR: 1–4) times, respectively. At the index colonoscopy, 7,671 (57.2%) subjects were categorized as normal group, 4,420 (32.9%) were categorized as low-risk group, and 1,335 (9.9%) were categorized as high-risk group. Subjects with normal group were approximately two years younger (58.7±9.8 years) than other groups (P<0.001). The normal group was comprised of significantly more females (58.5% vs 43.8% at low risk group; P<0.001, 58.5% vs 42.4% at high risk group; P<0.001). Rates of advanced adenoma at first surveillance colonoscopy were 2.7% (normal group at index colonoscopy), 5.5% (low-risk), and 8.8% (high-risk), with all of the groups being significantly different from each other (P<0.001).

**Table 1:** Characteristics of the study participants.

Index colonoscopy	Normal	Low risk	High risk	Total
	(n = 7,671)	(n = 4,420)	(n = 1,335)	(n = 13,426)
**Sex**				
Male	3,184 (41.5%)	2,486 (56.2%)	769 (57.6%)	6,439 (48.0%)
Female	4,487 (58.5%)	1,934 (43.8%)	566 (42.4%)	6,987 (52.0%)
**Age,** mean (SD)	58.7 (±9.8)	60.5 (±9.3)	60.5 (±9.6)	59.5 (±9.6)
<60	4,073 (53.1%)	1,942 (43.9%)	598 (44.8%)	6,613 (49.3%)
60–69	2,371 (30.9%)	1,640 (37.1%)	480 (36.0%)	4,491 (33.5%)
>70	1,227 (16.0%)	838 (19.0%)	257 (19.2%)	2,322 (17.2%)
**time period to first surveillance** (year), median (IQR*)	1.81 (1.08–3.03)	1.39 (1.03–2.36)	1.16 (1.00–1.94)	1.60 (1.04–2.76)
**first surveillance finding**				
Normal	5,583 (72.8%)	1,905 (43.1%)	621 (46.5%)	8,109 (60.4%)
Low risk	1,881 (24.5%)	2,271 (51.4%)	597 (44.7%)	4,749 (35.4%)
High risk	207 (2.7%)	244 (5.5%)	117 (8.8%)	568 (4.2%)
**Follow-up period after first surveillance** (year), median (IQR)	5.23 (2.96–8.32)	4.71 (2.74–7.64)	4.21 (2.57–6.59)	4.98 (2.85–7.94)
**Number of exam times after first surveillance colonoscopy**, median (IQR)	2 (1–4)	2 (1–4)	2 (1–4)	2 (1–4)

*IQR: Inter-Quartile Range

### Incidence of AN at second surveillance and subsequent colonoscopies based on the index and first surveillance colonoscopies

**Fig 2:**
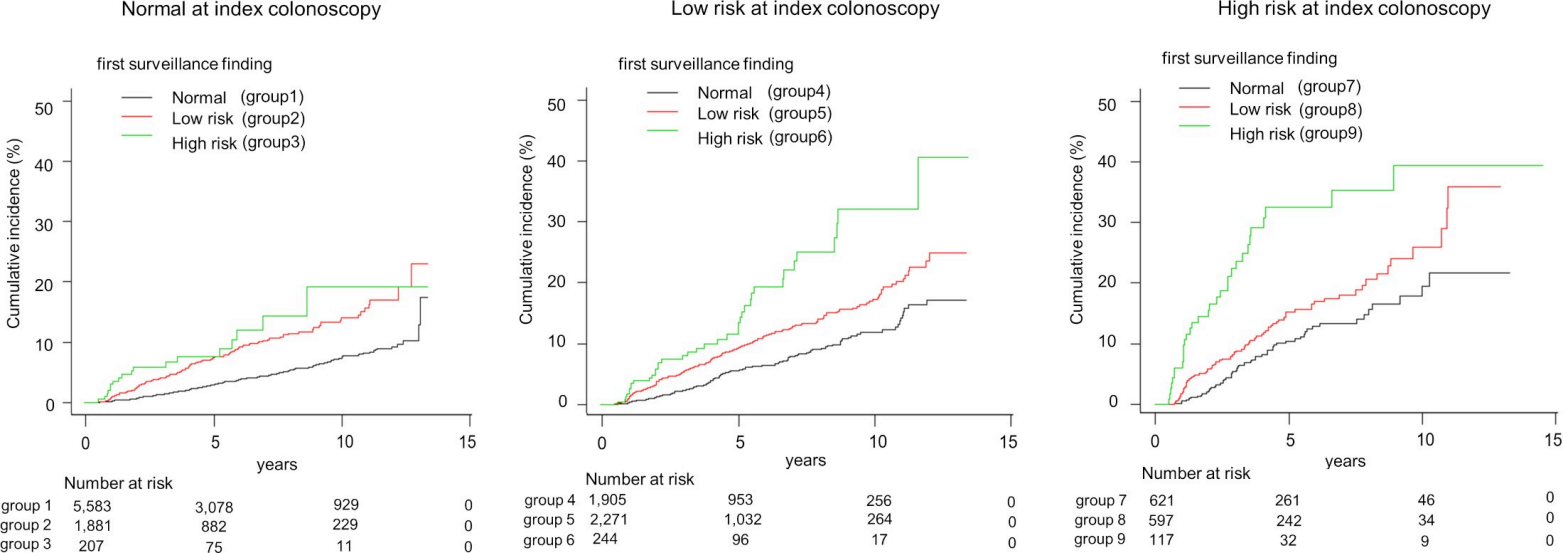
Cumulative Incidence of ANs according to each group after index and first surveillance colonoscopies.

**Fig 3:**
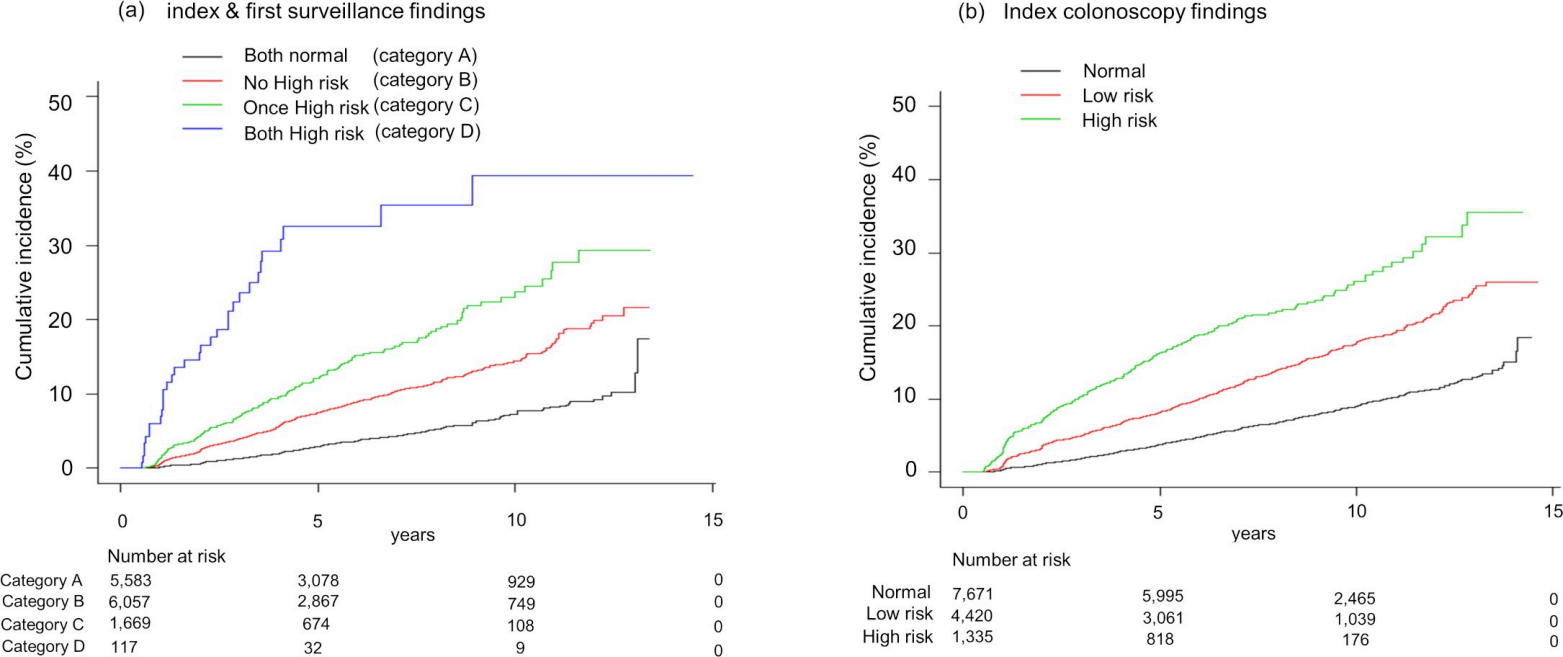
Cumulative Incidence of ANs in each Category by index and first surveillance colonoscopy. (a): Cumulative Incidence of AN after the first surveillance colonoscopy in each category. (b): Cumulative Incidence of AN after the index colonoscopy by the index colonoscopy findings alone.

The incidence of AN in each group based on the index and first surveillance colonoscopies, i.e. two consecutive colonoscopies, is shown in Table 2. AN was detected in 966 (7.2%) participants at the second or subsequent colonoscopies. In order to assess the effects of index colonoscopy and first surveillance colonoscopy, i.e. two consecutive colonoscopies, on subsequent AN finding, the patients were sub-categorized into nine groups (groups 1–9) as Table 2. In normal group at the index colonoscopy (groups 1–3), AN was found in 4.0% (225/5,583), 7.9% (148/1,881), and 8.7% (18/207) in each group. Similarly, in low risk group on the index colonoscopy (groups 4–6), AN was found in 6.6% (126/1,905), 10.1% (230/2,271), and 15.6% (38/244), respectively. In high risk group at the index colonoscopy (groups 6–9), AN was found in 10.1% (63/621), 14.4% (86/597), and 27.4% (32/117), respectively. In Fig 2, the Kaplan-Meier curve of the cumulative incidence of AN for each group (groups 1–9) after two consecutive colonoscopies is plotted. Based on the hazard ratio for each group in Table 2, the nine groups were divided into four categories: category A (both normal), category B (no high risk), category C (one high risk), category D (both high risk). The results of the Kaplan-Meier curves showed that there were significant differences among the four categories (P<0.001). This also showed that the findings of the index colonoscopy and the first surveillance colonoscopy were clearly associated with the incidence of AN, especially in category D (Fig 3).

**Table 2:** Incidence of advanced neoplasia after first surveillance.

Group	Index colonoscopy	first surveillance	N	ANs after first surveillance	HR	95% CI*	P value
1	Normal	Normal	5,583	225 (4.0%)	1		
2		Low risk	1,881	148 (7.9%)	2.27	1.85–2.80	<0.001
3		High risk	207	18 (8.7%)	3.22	2.00–5.20	<0.001
4	Low risk	Normal	1,905	126 (6.6%)	1.77	1.42–2.20	<0.001
5		Low risk	2,271	230 (10.1%)	2.97	2.47–3.56	<0.001
6		High risk	244	38 (15.6%)	5.26	3.73–7.42	<0.001
7	High risk	Normal	621	63 (10.1%)	3.14	2.38–4.16	<0.001
8		Low risk	597	86 (14.4%)	4.77	3.72–6.12	<0.001
9		High risk	117	32 (27.4%)	10.59	7.31–15.35	<0.001

*CI: Confidence interval

Group1; Normal at index & Normal at 1^st^ surveillance, group2; Normal & Low risk, group3; Normal & High risk, group4; Low risk & Normal, group5; Low risk & Low risk, group6; Low risk & High risk, group7; High risk & Normal, group8; High risk & Low risk, group9; High risk & High risk

A Cox regression analysis was performed on the index and first colonoscopy findings accounting for age and sex (Table 3). Based on the index colonoscopy findings alone, the hazard ratio in the high-risk group compared to the normal group was 3.42 (95% Confidence interval (CI) 2.95–3.96, P<0.001). On the other hand, the hazard ratio for AN of category D compared to category A was 9.90 (95% CI 6.82–14.35, P<0.001). The highest risk group, category D, was extracted by classification by indicators and results of initial surveillance colonoscopy.

**Table 3:** Clinical risk factors predicting ANs at follow-up colonoscopies using cox regression analysis.

Covariates	HR	95%CI		P value
		Lower	Upper	
**Index & first surveillance colonoscopy**				
Sex				
Female	1			
Male	1.18	1.04	1.34	0.012
Age	1.02	1.01	1.03	<0.001
Baseline findings				
Both normal (category A)	1			
No High risk (category B)	2.2	1.88	2.58	<0.001
Once High risk (category C)	3.75	3.1	4.55	<0.001
Both High risk (category D)	9.9	6.82	14.35	<0.001
**Index colonoscopy**				
Sex				
Female	1			
Male	1.26	1.13	1.4	<0.001
Age	1.02	1.02	1.03	<0.001
Baseline findings				
Normal	1			
Low risk	1.93	1.72	2.16	<0.001
High risk	3.42	2.95	3.96	<0.001

## Discussion

The United States of America has had great success with CRC screening, having decreased CRC mortality by more than half by effectively distributing the limited resource of colonoscopy [8]. To ensure adequate surveillance, both the mortality reduction effect of CRC and the strain on the limited availability of colonoscopy must be considered. To determine the optimal risk classification using the findings of two consecutive colonoscopies, the large cohort study enrolling 13 thousand subjects with the median follow up period of 7.52 years was conducted. The results of risk stratification based on the index colonoscopy in our study were similar to those of the US Multi-Society Task Force (US-MSTF) and the European Society of Gastrointestinal Endoscopy (ESGE) guidelines, even though we did not consider about the number of small polyps and did not resect some diminutive polyps which showed no advance features as shown in Table 3 and Fig 3 [2, 5]. Also, the combination of findings of the index and first surveillance colonoscopies for AN incidence gave the same trend of risk stratification. Moreover, the subjects with high-risk adenoma findings at both points (category D) had a much higher risk of AN, at 27.4%, than the other categories. We extracted highest risk group which would be examined closely. Conversely, the subjects with no adenoma findings at both points (category A) had the lowest risk results, at 4.0%. The hazard ratio of category D compared to category A was 9.90 (95% CI 6.82–14.35, P<0.001), suggesting that subjects who show advanced adenoma at both of two consecutive colonoscopies may still have a high probability of AN incidence on follow-up colonoscopies.

There are some studies that have investigated the usefulness of two previous colonoscopies in the prediction of the incidence of AN [9–14]. As per the Guideline by the US-MSTF, if the baseline colonoscopy showed advanced adenomas and the first surveillance examination showed no adenoma, low-risk adenomas, or high-risk adenomas, the recommended intervals for the next examinations were 5 years, 5 years, and 3 years, respectively. The Guideline has provided updated recommendations for surveillance based on the relationship of the baseline and first surveillance findings. Furthermore, more evidence is needed to set the best intervals for surveillance [2]. Based on the Kaplan-Meier analysis of the present study, when a cumulative incidence of 10% was set as the acceptable frequency, the acceptable interval was approached at 12.3 years in category A, at 6.8 years in category B, at 4.2 years in category C, and at 1.1 year in category D. The present findings suggest that category D needs more careful surveillance with shorter intervals. On the other hand, a longer follow-up than suggested so far would be accepted in category A. With our results, a strategy with two consecutive colonoscopies findings would be one of solutions for adequate surveillance.

The main aim of surveillance endoscopy is to reduce post-colonoscopy colorectal cancer (PCCRC) and CRC deaths. In this study, AN was used as a surrogate endpoint for precursors of CRCs, because PCCRCs are rare events. Some studies have examined the impact of surveillance on the incidence of long-term CRC and the incidence of CRC after adenoma resection [8, 15, 16]. PCCRC have been reported 0.38–2.4 cancers per 1000 person-years [17]. Majority of PCCRCs can arise from missed or incompletely resected lesions [18, 19]. Although the quality of colonoscopy largely influences the incidence of PCCRC, the quality of the procedure (bowel preparation level, adenoma detection rate, etc.) was not examined in this study. At the index colonoscopy, we detected adenoma or colorectal cancer in 43.1% (n = 5,783/13,426) and AN in 10.2% (n = 1,363/13,426). The PCCRCs was detected 0.95 cancers per 1000 person-year at the first surveillance colonoscopy in this study, and 78.5% (10,524/13,426) of the subjects had their first surveillance colonoscopy within 3 years. PCCRCs are recognized as a critical quality indicator, therefore qualities in the present study would be acceptable.

In a resource-constrained setting, it is important to consider the cost-effectiveness of conducting surveillance. On the other hands, the first surveillance colonoscopy with a short interval would have the potential for early detection of PCCRC. Some of PCCRC were detected on subjects with normal findings at index colonoscopy. A strategy of risk stratification based on two consecutive colonoscopies even with subjects with normal findings at index colonoscopy would be acceptable in some countries with different health care environments (cost, capacity, etc.).

The ESGE and the US-MSTF guidelines recommend that 5 or more diminutive adenomas should be repeat colonoscopy at 3–5 years intervals. In this study, not all diminutive polyps were resected, even they were diagnosed as adenomatous lesions. For this reason, we did not consider that the number of polyps in the risk stratification. These polyps may have the potential of becoming larger or progressing to an advanced adenoma. However, our study showed same trend as the western guidelines, even if we did not consider about diminutive polyps [2, 5]. There are some reports that the rate of invasive carcinoma within diminutive adenomas was 0.03–0.3% [20, 21]. Ninomiya et al. reported that 706 patients with diminutive polyps were follow-up for 81.4±15.8 months, and five polyps were considered to have grown into lesions to be resected (colorectal neoplasia ≥6 mm, depressed lesions, and lesions with a type V pit pattern) [22].

The present study has some important implications for adenoma surveillance. Its first strength is the clinical setting in which this study was conducted (i.e., “real-world” clinical long-term practice) as opposed to a clinical trial setting. In the present study, the median observation period for participants was 7.5 (IQR: 5.1–10.4) years since the index colonoscopy. Surveillance procedures were performed at a variety of time intervals, suggesting that the present results are potentially more representative of community-based practice in Japan. The nationwide survey reported that most Japanese institutions preferred shorter intervals than the recommendations by the US-MSTF and ESGE [23]. Thus, it could be said that the present study reflects the present colonoscopy environment in Japan. Second, the present study had a large sample size. There are several studies that performed a similar analysis, but the number of eligible subjects has ranged from a few hundred to several thousand. On the other hand, more than 10,000 subjects were analyzed in the present study. In addition, the present study proposed well-divided risk groups, with fairly-precise proportions of subsequent risks for AN incidence. Furthermore, a large number of subjects with normal index colonoscopy findings was included, with category A accounting for 41.6% of all subjects. Since category A represents approximately half of all cases, unnecessary colonoscopy should be decreased after two consecutive colonoscopies.

There were some limitations in the present study. First, it was a retrospective cohort study, and data were collected at two endoscopic clinics. However, the large number of study subjects would dilute selection bias, and many endoscopists participated. Second, there was no clinical information on other candidate risk factors (e.g., smoking, alcohol intake, medication, body mass index, familial history). Although they are important factors in the development of the lesion, endoscopic findings are the factors that strongly affect the risk of subsequent CRC development [24–26]. Third, the importance of serrated lesions has been reported in recent researches. High risk metachronous serrated lesions have been found at surveillance colonoscopy in subjects with high risk serrated lesions and higher age at first index colonoscopy [27, 28]. We could not consider the importance of serrated lesions, because some small serrated lesions without advanced features left unresected as well as diminutive polyps and pathologic diagnosis of differentiation between non-significant hyperplastic polyp and sessile serrated polyps was not established in the former half of the study period. So, we applied the findings of dysplasia only.

## Conclusion

Classification based on the findings of index and first surveillance colonoscopies more effectively stratified the risk of subsequent AN, resulting in more proper allocation of limited colonoscopy resources with more appropriate surveillance interval after two consecutive colonoscopies.

## Supporting information

S1 File Data of all patients for the figures and the tables. (CSV)
